# Effects of sarcopenia and sarcopenic obesity on joint pain and degenerative osteoarthritis in postmenopausal women

**DOI:** 10.1038/s41598-022-17451-1

**Published:** 2022-08-08

**Authors:** Hye In Kim, So Hyun Ahn, Yup Kim, Ji Eun Lee, Euna Choi, Seok Kyo Seo

**Affiliations:** 1 Department of Obstetrics and Gynecology, Severance Hospital, Yonsei University College of Medicine, 50 Yonsei-ro, Seodaemun-gu, Seoul, 03722 Republic of Korea; 2 Institute of Women’s Life Medical Science, Yonsei University College of Medicine, Seoul, Republic of Korea

**Keywords:** Disease prevention, Geriatrics, Risk factors, Bone, Muscle

## Abstract

This study aimed to identify the prevalence of sarcopenia, obesity, and sarcopenic obesity and examine their association with radiographic knee osteoarthritis (OA) and knee pain in Korean postmenopausal women. This cross-sectional study utilized the data from Korean National Health and Nutrition Examination Surveys 2009–2011. The participants were categorized into 4 groups based on body composition: either sarcopenic (appendicular skeletal muscle < 23%) or not, either obese (body mass index ≥ 25.0 kg/m^2^) or not. The prevalence of radiographic knee OA and knee pain was calculated. The effect of hormone replacement therapy (HRT) was also evaluated. The prevalence of radiographic knee OA, knee pain, and both were all highest in the sarcopenic obese group and lowest in the control group (61.49% vs. 41.54%, 39.11% vs. 27.55%, 32.04% vs. 17.82%, all p < 0.001). Without sarcopenia, obese women showed significantly higher ratio of radiographic knee OA only (57.64% vs. 41.54%, p < 0.001). With sarcopenia, the coexistence of obesity presented higher ratio of radiographic knee OA, knee pain, and both compared to sarcopenia without obesity (61.49% vs. 41.82%, 39.11% vs. 27.61%, 32.04% vs. 17.60%, all p < 0.001). The use of HRT for more than 1 year was not associated with radiographic knee OA, knee pain, or both (p = 0.147, 0.689 and 0.649, respectively). Obesity with sarcopenia had greater effect on knee OA compared to obesity without sarcopenia. Moreover, HRT use for more than 1 year was not associated with the prevalence of knee OA. Therefore, more efforts should focus on reducing body fat and increasing muscle in postmenopausal women with knee OA.

## Introduction

Osteoarthritis (OA) is a common disease in the elderly population, affecting approximately 27 million adults in the United States^1^. OA often requires surgical treatment owing to pain and mobility restriction, and the social cost is high due to high postoperative morbidity^2^. Therefore, it is important to clarify the pathophysiology and reduce risk factors. OA and its various risk factors have been extensively studied in the general elderly population; however, studies in postmenopausal women are limited.

Menopause is a life event that greatly affects women through hormonal changes. The most well-known menopausal symptoms include vasomotor symptoms including hot flushes, atrophic vaginitis, psychological symptoms including depression, and cardiovascular symptoms^3^. Furthermore, temporomandibular joint disease, osteoporosis, and sarcopenic obesity have a significantly higher incidence in women than in men, suggesting a relationship between female hormones and these musculoskeletal ailments^4–6^. In addition, a change in hormonal status, especially a decrease in sex hormone levels, alters the body composition^7^. A decrease in bone mass density leads to osteoporosis, and the decrease in muscle mass leads to sarcopenia increases^8^. All these changes lead to a restricted life in postmenopausal women^9^.

Therefore, it is necessary to examine the relationship between body composition and OA in postmenopausal women. Previous studies primarily focused on the association between obesity and OA. Furthermore, the research was conducted based on anthropometric measures such as weight and BMI. However, BMI does not reflect body composition as do body fat mass and muscle mass^10,11^. Sarcopenia, which is characterized by a decrease in muscle mass, is a part of normal aging process but it results in functional impairment^12^. In postmenopausal women, it is rather necessary to utilize objective measures for relatively low muscle mass to study sarcopenia and sarcopenic obesity in relation to OA^13^.

Because low muscle mass may be owing to a deprivation of sex hormone levels in postmenopausal women, estrogen supplementation or hormone replacement therapy (HRT) is used to relieve various symptoms and to protect muscle mass and muscle strength^14,15^; however, a few studies showed that HRT did not show positive effect on muscle mass^16,17^. Furthermore, the effect of HRT on knee OA is controversial; although some observational studies showed positive effect of HRT on knee OA^18,19^, others showed no association of sex hormone replacement or knee arthroplasty^20^.

Therefore, this study aimed to identify the prevalence of sarcopenia, obesity, and sarcopenic obesity and examine their association with knee OA in Korean postmenopausal women and also the effect of HRT using data obtained during the 2009–2011 Korean National Health and Nutrition Examination Surveys (KNHANES).

## Methods

### Study design and setting

This was a nationwide cross-sectional observational study. Data were extracted from the KNHANES conducted from 2009 to 2011^21^. KNHANES is a national, population-based cross-sectional survey that collects data on the health and nutrition status of Koreans by sampling representative, noninstitutionalized Korean civilians. Every year, approximately 10,000 individuals were sampled. The survey is composed of health interviews, physical examinations, and nutrition surveys. All the components of the survey were conducted either at the mobile screening center or at the home of participants by trained medical staff, dieticians, health interviewers, and medical technicians. All participants provided written informed consent. The KNHANES were performed in accordance with the principles of the Declaration of Helsinki. For this study, we used the data from 2009 to 2011; the study was approved by the institutional review board of the Korea Centre for Disease Control and Prevention (2009-01CON-03-2C, 2010-02CON-21-C, 2011-02CON-06C).

### Participants

For KNHANES from 2009 to 2011, 28,009 individuals were enrolled. Of the participants, 4362 women answered as postmenopausal for the question about their menstruation status. We included only the participants who completed health interviews for sarcopenia, physical examination for BMI, knee joint radiography, and dual-energy X-ray absorptiometry (DXA). The exclusion criteria included diagnosis of surgical menopause, thyroid disease, end-stage renal disease, malignant tumor, or arthritis other than OA and missing variables for analysis. For sub-cohort analysis, we excluded women who did not respond to the questions regarding hormone replacement therapy (HRT). The participants were asked about the experience of HRT and the period of use in months. For this study, 4099 women were included in the final analysis. Among those who answered to have the history of HRT use, they were divided into the group who had used HRT for less than a year, whereas the other who had used it for longer than a year for sub-cohort analysis.

### Definition of sarcopenia and obesity

Appendicular skeletal muscle (ASM) was defined as the sum of muscle mass in four limbs as measured by DXA (QDR 4500A; Hologic Inc., Bedford, MA). For the definition of sarcopenia, there is no single standardized diagnostic criterion. Based on a previous study that studied for sarcopenia and sarcopenic obesity using the KNHANES from 2010 to 2011, we used the value obtained by dividing ASM by body weight multiplied by 100 as the ASM index (%). Sarcopenia was defined as two standard deviations below the mean of the same gender, young standard group and the cutoff value was 23.0% for Korean women^22^. Obesity was defined as body mass index (BMI, kg/m^2^) more than or equal to 25.0 according to the guideline for the management of obesity in Korea^23^.

### Measurements of variables

Current age, age at menarche and menopause, body mass index (BMI), past history of hypertension (HTN), diabetes mellitus (DM), osteoporosis, lifestyle such as alcohol consumption and smoking status, HRT, and lipid profile were considered as confounding variables.

All participants completed the physical examination and blood sampling either at the mobile screening center or at home. They wore light clothing without shoes while measuring the height, weight, and blood pressure. BMI was calculated from the measured height and weight. Blood sampling was done after overnight fasting of at least 8 h and the samples were analyzed on the same day (Neodin Medical Institute, Seoul, South Korea).

All participants completed the standardized questionnaire regarding their medical history and lifestyle. For medical history, prior to doctor’s diagnosis of HTN, DM, and osteoporosis were asked. For alcohol consumption, any previous experience was asked. A heavy drinker was defined as he/she who consumed more than 30 g of alcohol per day. For smoking, the participants were asked of any previous experience of smoking and the current status of smoking. Regular exercise was defined as walking for 30 min or more at a time, 5 days per week or more and muscle-strengthening exercise as anaerobic exercises such as push-ups, sit-ups, dumbbells, and barbells at least once a week. Regarding HRT, the history of drug use was asked. The duration was measured in months. If they had used HRT for less than a year, they were assigned to sub-cohort 1, and if they had used HRT for longer than a year, they were assigned to sub-cohort 2.

Knee radiography was performed for all participants and the results were analyzed based on the Kellgren-Lawrence (KL) Scale that assesses the severity of knee OA. A grade higher than or equal to 2 was considered as radiographic knee OA. In addition, knee pain was asked on the questionnaire as knee pain for 30 days over the previous 3 months.

### Statistical analysis

Statistical analysis software (SAS) version 9.4 (SAS Inc., Cary, NC, USA) was used for all statistical analysis and the KNHANES data were analyzed according to the KNHANES data analysis guidelines. All analyses were two tailed, and a p-value of < 0·05 was considered significant.

Continuous variables were analyzed by one-way ANOVA considering the complex sample design to calculate the mean and standard errors (SE). Categorical variables were represented with raw percent with SEs and comparatively tested by Rao-Scott Chi-square test.

For the sub-cohort analysis, the participants were divided based on the period of HRT consumption. After adjusting for age and BMI, the continuous variable was represented with mean and SE, and analysis of covariance was applied to test statistical significance. The dichotomous variables were represented with ratio and standard deviation (SD), and logistic regression model was used to identify association.

## Results

Subjects were divided into four groups based on their body composition: (1) control: did not meet the definition for obesity or sarcopenia; (2) sarcopenic: met definition for sarcopenia but not obesity; (3) obese: met definition for obesity but not sarcopenia; (4) sarcopenic obese: met definition for both sarcopenia and obesity.

### Demographic characteristics of participants

From 2009 to 2011, 28,009 people participated in KNHANES, of which 4362 participants were postmenopausal. Our final cohort included 4150 subjects after excluding those who did not complete the survey. 1231 people were with normal body composition, 1379 were sarcopenic but not obese, 203 were obese but not sarcopenic, and 1337 were both sarcopenic and obese. The baseline characteristics of participants according to the body composition are presented in Table 1.

**Table 1:** Baseline clinical characteristics of all subjects.

	Total (N = 4150)		Control (N = 1231)		Sarcopenic (N = 1379)		Obese (N = 203)		Sarcopenic obese (N = 1337)		p-value
	Mean or row percent	Standard error	Mean or row percent	Standard error	Mean or row percent	Standard error	Mean or row percent	Standard error	Mean or row percent	Standard error	
Age (year)	62.41	0.25	62.79	0.49	62.20	0.41	60.20	1.04	62.60	0.36	0.098
Age at menopause (year)	48.40	0.12	47.80	0.25	48.54	0.21	47.93	0.63	48.84	0.21	0.012
Age at menarche (year)	13.96	0.28	14.69	0.51	13.78	0.51	12.53	0.97	13.71	0.38	0.152
Height (cm)	153.47	0.14	153.92	0.30	153.38	0.23	154.17	0.63	153.05	0.20	0.066
Weight (kg)	57.15	0.18	51.40	0.28	53.91	0.20	62.98	0.59	64.91	0.25	< 0.001
BMI (kg/m^2^)	24.23	0.07	21.64	0.08	22.88	0.05	26.45	0.12	27.67	0.09	< 0.001
Waist circumference (cm)	82.33	0.22	75.75	0.27	79.46	0.25	87.33	0.69	90.54	0.33	< 0.001
ASM index (%)	24.65	0.08	27.64	0.08	23.60	0.06	26.65	0.07	22.77	0.07	< 0.001
**Past history**											
Hypertension											< 0.001
No	60.10	1.11	73.25	1.93	61.23	1.90	64.79	5.04	46.53	2.01	
Yes	39.90	1.11	26.75	1.93	38.77	1.90	35.21	5.04	53.47	2.01	
Diabetes mellitus											0.001
No	87.30	0.73	90.63	1.06	88.03	1.17	84.96	3.53	83.92	1.28	
Yes	12.70	0.73	9.37	1.06	11.97	1.17	15.04	3.53	16.08	1.28	
Osteoporosis											0.927
No	81.40	0.86	80.59	1.56	81.58	1.41	82.59	3.34	81.76	1.61	
Yes	18.60	0.86	19.41	1.56	18.42	1.41	17.41	3.34	18.24	1.61	
**Drinking**											
Alcohol consumption											0.855
No	31.65	1.12	32.90	1.90	30.79	1.73	31.15	4.22	31.50	1.86	
Yes	68.35	1.12	67.10	1.90	69.21	1.73	68.85	4.22	68.50	1.86	
Amount of alcohol/time											0.227
Mild to moderate	67.04	1.57	67.01	2.98	69.55	2.46	73.26	4.90	63.27	2.82	
Heavy (> 30 g/day)	32.96	1.57	32.99	2.98	30.45	2.46	26.74	4.90	36.73	2.82	
**Smoking**											
Smoking											0.268
Never smoked	90.44	0.74	88.95	1.23	89.93	1.29	91.35	3.15	92.17	1.06	
Current/past smoker	9.56	0.74	11.05	1.23	10.07	1.29	8.65	3.15	7.83	1.06	
Current status of smoking											0.171
Past smoker	46.06	3.99	38.66	5.63	43.65	6.66	53.23	14.51	57.38	7.22	
Current smoker	53.94	3.99	61.34	5.63	56.35	6.66	46.77	14.51	42.62	7.22	
**Exercise**											
Regular exercise											0.004
No	87.50	0.82	86.61	1.47	90.67	1.08	81.87	3.44	85.79	1.34	
Yes	12.50	0.82	13.39	1.47	9.33	1.08	18.13	3.44	14.21	1.34	
Muscle-strengthening exercise											0.187
No	85.41	0.84	82.99	1.59	85.82	1.32	85.71	3.07	87.09	1.37	
Yes	14.59	0.84	17.01	1.59	14.18	1.32	14.29	3.07	12.91	1.37	
**HRT**											
History of HRT use (%)											0.760
No	84.23	0.82	83.57	1.59	83.83	1.25	83.21	3.41	85.39	1.35	
Yes	15.77	0.82	16.43	1.59	16.17	1.25	16.79	3.41	14.61	1.35	
Duration of HRT use (%)											0.516
< 1 year	37.97	2.45	33.20	4.92	41.85	3.87	30.39	11.52	39.51	4.67	
≥ 1 year	62.03	2.45	66.80	4.92	58.15	3.87	69.61	11.52	60.49	4.67	
Duration of HRT use (month)	31.68	1.97	35.05	4.36	28.10	2.59	24.55	3.93	33.63	3.81	0.381

Sarcopenic: ASM index < 25.6%; Obese: BMI ≥ 25.

Continuous variables were analyzed by one-way ANOVA and categorical variables were analyzed by Rao-Scott Chi-square test.

*BMI* body mass index, *ASM* appendicular skeletal muscle mass, *Wt* body weight, *HRT* hormone replacement therapy.

The mean age of the participants was 62.41 ± 0.25 years. The mean age at menopause and menarche was 48.40 ± 0.12 years and 13.96 ± 0.28 years, respectively. Only the mean age at menopause was significantly different among the groups (p = 0.012). The characteristics related to obesity such as weight, body mass index (BMI), and waist circumference, and ASM index presented significant difference among the four groups in the expected direction (p < 0.001).

For the past history, the prevalence of HTN and DM was significantly different among the four groups with the prevalence being the highest for the sarcopenic obese group for both HTN and DM (p < 0.001), while that of osteoporosis was not significantly different (p = 0.927).

In terms of lifestyle, smoking and alcohol consumption were not significantly different among the groups. The two obese groups tended to exercise regularly more than the non-obese women (p = 0.004). The previous use of HRT was not significantly different among the groups (p = 0.760).

### Radiographic and clinical knee OA based on body composition

Table 2 shows the prevalence of radiographic knee OA and knee pain of the four groups. The ratio of radiographic knee OA, knee pain, and both radiographic knee OA and knee pain were analyzed. There was a significant difference among the four groups in all three variables (all p < 0.001).

**Table 2:** Prevalence of radiographic knee OA, knee pain and both radiographic and knee pain in each group.

	Total (N = 4150)		Control (N = 1231)		Sarcopenic (N = 1379)		Obese (N = 203)		Sarcopenic obese (N = 1337)		Overall p-value
	Mean or row percent	Standard error	Mean or row percent	Standard error	Mean or row percent	Standard error	Mean or row percent	Standard error	Mean or row percent	Standard error	
**Radiographic knee OA**											< 0.001
Yes	48.75	1.18	41.54	2.10	41.82	2.09	57.64	4.99	61.49	2.01	
No	51.25	1.18	58.46	2.10	58.18	2.09	42.36	4.99	38.51	2.01	
**Knee pain**											< 0.001
Yes	31.41	1.27	27.55	2.21	27.61	1.96	29.91	5.22	39.11	2.22	
No	68.59	1.27	72.45	2.21	72.39	1.96	70.09	5.22	60.89	2.22	
**Radiographic knee OA & knee pain**											< 0.001
Yes	22.70	1.12	17.82	1.93	17.60	1.70	25.96	5.17	32.04	2.20	
No	77.30	1.12	82.18	1.93	82.40	1.70	74.04	5.17	67.96	2.20	

Radiographic knee OA, Kellgren–Lawrence grade scale grade ≥ 2; Knee pain, knee pain for 30 days over the past 3 months.

Continuous variables were analyzed by one-way ANOVA and categorical variables were analyzed by Rao-Scott Chi-square test.

*OA* osteoarthritis.

Table 3 shows the p-value as a result of multiple groupwise comparisons among the four groups. Among the non-obese people, whether sarcopenic or non-sarcopenic did not show significant difference in the ratios of each and both radiographic OA and knee pain (p = 0.922, 0.981, and 0.932, respectively). Also, among the obese people, the ratios of each and both radiographic OA and knee pain did not show significant difference between the non-sarcopenic and the sarcopenic group (p = 0.467, 0.118, and 0.298, respectively).

**Table 3:** Multiple comparison results of the prevalence of radiographic knee OA, knee pain and both radiographic and knee pain between each two groups of non-obese, non-sarcopenic, sarcopenic and obese people.

	Non-obese	Non-sarcopenic	Sarcopenic	Obese
	Non-sarcopenic vs. sarcopenic	Non-obese vs. obese	Non-obese vs. obese	Non-sarcopenic vs. sarcopenic
Radiographic knee OA	0.922	< 0.001	< 0.001	0.467
Knee pain	0.981	0.658	< 0.001	0.118
Radiographic knee OA & knee pain	0.932	0.098	< 0.001	0.298

Radiographic knee OA, Kellgren-Lawrence grade scale grade ≥ 2; Knee pain, knee pain for 30 days over the past 3 months.

Post-hoc corrections of ANOVA by Bonferroni’s method was done; p-value smaller than 0.0083 is statistically significant.

*OA* osteoarthritis.

However, among the people without sarcopenia, the obese people tended to show a higher ratio of radiographic OA significantly (57.64% vs. 41.54%, p < 0.001). Knee pain and both radiographic and clinical knee OA were not significantly different (p = 0.658 and 0.098, respectively). Finally, for people with sarcopenia, obesity increased the ratio of radiographic knee OA, knee pain, and both radiographic knee OA and knee pain. For radiographic knee OA, although the ratio was 41.82% for the sarcopenic group, that of the sarcopenic obese group was 61.49% (p < 0.001). The ratio of knee pain was also statistically different between the sarcopenic and sarcopenic obese groups (27.55% and 39.11%, respectively; p < 0.001). Lastly, the ratio of people with both radiographic knee OA and knee pain was 17.60% and 32.04% for sarcopenic and sarcopenic obese groups, respectively (p < 0.001).

### Hormone replacement therapy

Based on the answers to the use of HRT in the questionnaire, a sub-cohort analysis was done. Among the postmenopausal women who had used HRT before, 240 women used HRT for less than a year, whereas the other 398 used it for longer than a year. In Table 4, the mean ASM index of these two groups did not show a significant difference (24.47% and 24.80%, respectively, p = 0.181). The ratio of sarcopenia showed no significant association with the duration of HRT use (69.80% and 62.11%, respectively, p = 0.147). For the radiographic knee OA, knee pain, and both radiographic knee OA and knee pain, none of the ratios were significant between the two groups (p = 0.688, 0.634, and 0.649, respectively).

**Table 4:** ASM index, prevalence of radiographic knee OA, knee pain and both radiographic and knee pain of the women with history of HRT use.

	Duration of HRT use < 1 year (N = 240)		Duration of HRT use ≥ 1 year (N = 398)		p-value
	Mean or row percent	Standard error	Mean or row percent	Standard error	
ASM index (%, mean)	24.47	0.20	24.80	0.16	0.181
**Sarcopenia (proportion)**					0.147
No	30.20	4.02	37.89	3.24	
Yes	69.80	4.02	62.11	3.24	
**Radiographic knee OA (proportion)**					0.689
No	62.27	4.36	64.47	3.28	
Yes	37.73	4.36	35.53	3.28	
**Knee pain (proportion)**					0.634
No	75.23	4.11	77.73	3.38	
Yes	24.77	4.11	22.27	3.38	
**Radiographic knee OA & knee pain (proportion)**					0.649
No	86.84	3.30	88.64	2.14	
Yes	13.16	3.30	11.36	2.14	

Radiographic knee OA, Kellgren–Lawrence grade scale grade ≥ 2; knee pain, knee pain for 30 days over the past 3 months.

Continuous variables were analyzed by one-way ANOVA and categorical variables were analyzed by Rao-Scott Chi-square test.

*OA* osteoarthritis, *ASM* appendicular skeletal muscle mass, *Wt* body weight, *HRT* hormone replacement therapy.

Table 5 shows the results after adjustment for age and BMI. The average ASM index was not significantly different between the two groups (p = 0.119), which was similar to the result before the adjustment. Both the ratio of sarcopenia and radiographic OA did not show a significant difference between the two groups (p = 0.082 and p = 0.506, respectively).

**Table 5:** ASM index, prevalence of sarcopenia, radiographic knee OA of the women with history of HRT use (adjusted for age and BMI).

	Duration of HRT use < 1 year (N = 240)		Duration of HRT use ≥ 1 year (N = 398)		p-value*
	Mean or proportion*	Standard error	Mean or proportion*	Standard error	
ASM index (%, mean)	24.35	0.18	24.68	0.15	0.119
Sarcopenia (proportion)	0.77	0.05	0.67	0.08	0.082
Radiographic knee OA (proportion)	0.44	0.05	0.4022	0.0362	0.5061

Radiographic knee OA, Kellgren-Lawrence grade scale grade ≥ 2.

Continuous variables were analyzed by one-way ANOVA and categorical variables were analyzed by Rao-Scott Chi-square test.

*OA* osteoarthritis, *ASM* appendicular skeletal muscle mass, *Wt* body weight, *HRT* hormone replacement therapy.

*Adjusted mean or proportion and p-value were adjusted for age and BMI.

## Discussion

Menopause, which involves a drastic change in hormonal status and results in a decrease in sex hormone levels, has a great effect on bone mass density and body fat distribution^7^. A decrease in bone mass density can lead to osteoporosis, an increase in body fat to obesity, and a decrease in body muscle to sarcopenia, which are all closely related to knee OA in postmenopausal women. Additionally, HRT after menopause, which keeps the estradiol level enough to relieve menopausal symptoms, is considered to be helpful in preventing knee OA^18^.

Although the association between knee OA and anthropometric measures such as BMI and weight have been studied well, there are few studies regarding the association of body composition with knee OA in literature. However, the results of the studies were not in the same trend because of their different study design, participants, measurement of body composition, and definition of obesity^13,24–26^. The longitudinal study conducted by Misra et al. showed that the risk of knee OA was increased in sarcopenic obese people, especially in women with statistical significance^26^. However, another study showed that body weight and BMI were more important factors associated with knee OA compared to body composition such as fat distribution or muscle mass as measured by DXA^13^. In a study by Suh et al. that also utilized data from KNHANES, obese people with low muscle mass, who were sarcopenic obese, presented greater odds of radiographic knee OA compared to the non-sarcopenic non-obese people, whereas obese people without low muscle mass showed no increased odds^25^.

This is the first cross-sectional study with a large number to present the association between knee OA and body composition acquired with DXA such as sarcopenia and sarcopenic obesity. Based on the body composition analyzed with DXA, the participants were categorized into four categories. For people without sarcopenia, the status of obesity only affected the prevalence of radiographic knee OA. However, for people with sarcopenia, the status of obesity affected the prevalence of not only radiographic knee OA but also knee pain. This emphasizes the fact that obesity may have more effect only when sarcopenia coexists, suggesting that the risk of knee OA in postmenopausal women is through both high fat mass and low muscle mass, not just one. The contrasting results of a previous study by Misra et al. can be explained by the fact that Misra et al. included both sexes in their study, whereas we analyzed only postmenopausal women^26^.

Aging causes many changes in body composition. Usually, muscle mass and bone mass decrease and fat mass increases. The increased fat mass infiltrates into muscle and bone. This can be observed more clearly in postmenopausal women, especially as central obesity increases as estrogen decreases. Central obesity also means an increase in visceral fat^8,27,28^. As aging progresses, osteoporosis, sarcopenia, and obesity increase all together. As a result, the quality of life of postmenopausal women or mortality increases^29^. Previous literature has shown that sarcopenia is more prevalent in older women with OA^30^. According to one review, these two diseases are closely related in both clinical and cellular ways^31^. Clinically, lower limb muscle weakness can lead to OA, and conversely. Poor muscle quality and low lean body mass mechanically contribute to the pathogenesis of OA and, the walking speed, one of the functional diagnostic criteria for sarcopenia, is slower in the OA group^32,33^. In cellular pathway, chondrocytes and skeletal muscle cells share common pathological pathway, thus, muscle mass in most OA patients is reduced^31^. Therefore, it can be considered that not simply the increase in fat mass, but the decrease in muscle mass accompanying the increase in fat mass has a significant effect on knee OA.

HRT is used by postmenopausal women to relieve various menopausal symptoms such as hot flushes. HRT affects not only the vasomotor symptoms but also the prevalence of OA^19^. Cirillo et al. showed that neither estrogen replacement nor estrogen plus progestogen therapy affected the need for knee joint replacement^34^. Our study also included a sub-cohort analysis to see the long-term effect of HRT and we divided the participants based on the use of HRT for longer than a year^35^. Different from the studies that presented that HRT relieved both the prevalence of radiographic knee OA and knee pain, our results showed no statistical significance in comparison based on HRT^36,37^. Confounding factors such as duration, dose, type of HRT used, lifestyle, and daily activity could explain the discordant results.

This study has some strengths. First, this study utilized the standardized nationwide data with a great number of participants. Second, to see the effect of sarcopenia and obesity in relation to each other, we categorized the participants into four groups and performed group-to-group multiple comparisons. Third, considering the specificity of the participants, we also conducted a sub-cohort analysis to see the effects of HRT on both clinical and radiological knee OA.

However, this study has several limitations. First, the results of this comparative, cross-sectional study design cannot be interpreted as a causal relationship between body composition and knee OA. Second, the variables obtained by self-questionnaire are subject to information bias or recall bias. Third, sarcopenia was only defined with the low muscle mass. Although sarcopenia is now diagnosed with many functional values such as hand grip strength or physical performance, this study only utilized the muscle quantity. Because this study was planned before the consensus about the sarcopenia diagnosis and KNHANES only contained the data about muscle mass, further study with muscle quality should better support this result. Fourth, the type of HRT was not analyzed due to the lack of data. Further prospective studies with a large number of subjects are required for a more detailed explanation regarding the relationship between sarcopenic obesity and knee OA.

In conclusion, obesity in terms of sarcopenia has greater effect on knee OA compared to obesity without sarcopenia. Moreover, HRT use of longer than a year is not associated with the prevalence of knee OA. Therefore, in knee OA, more preventive efforts should focus on reducing body fat and increasing muscle in postmenopausal women.

## Data Availability

The datasets used and/or analyzed during the current study are available from the corresponding author on reasonable request.
